# Therapeutic Convergence in Neurodegeneration: Natural Products, Drug Repurposing, and Biomolecular Targets

**DOI:** 10.3390/biom15091333

**Published:** 2025-09-18

**Authors:** Caterina Vicidomini, Giovanni N. Roviello

**Affiliations:** Institute of Biostructures and Bioimaging, Italian National Council for Research (IBB-CNR), Area di Ricerca Site and Headquarters, Via Pietro Castellino 111, 80131 Naples, Italy; caterina.vicidomini@ibb.cnr.it

Neurodegenerative diseases pose an escalating global health burden, caused by their intricate pathophysiological mechanisms, and consequently, a persistent lack of curative therapies. A detailed exploration of these concepts, and of those presented below in this work, can be found in the Special Issue “Biomolecular Approaches and Drugs for Neurodegeneration”, available at https://www.mdpi.com/journal/biomolecules/special_issues/O40SG4E9N5 (accessed on 30 August 2025). Importantly, recent advances in molecular neuroscience have elucidated key processes underlying disorders such as Alzheimer’s disease, Parkinson’s disease, prion disorders, multiple sclerosis, and spinocerebellar ataxia type 1 [1,2,3]. These conditions share common features including selective neuronal vulnerability, dysregulated lipid metabolism, mitochondrial dysfunction, neuroinflammation, and protein misfolding. Emerging prophylactic [4] and therapeutic strategies increasingly target these converging pathways, with the latter leveraging both natural compounds and synthetic agents to restore cellular homeostasis and mitigate neurodegeneration [5,6,7,8,9]. Notably, neuropeptides play a multifaceted role in the pathophysiology of Alzheimer’s disease [10]. Alterations in their expression and receptor activity have been observed in affected brain regions, particularly the hippocampus and entorhinal cortex. These molecules contribute to neuroprotection by modulating synaptic plasticity, reducing beta-amyloid accumulation, enhancing glucose metabolism, and regulating stress responses such as endoplasmic reticulum stress and autophagy. Their involvement in key signaling pathways suggests that neuropeptides may serve not only as biomarkers of disease progression, but also as promising therapeutic targets for intervention in Alzheimer’s disease [10,11]. Complementing these molecular insights, recent findings have highlighted the role of microglial responses to myelin debris in aging brains, which contribute to cholesterol ester accumulation, a process implicated in neurodegenerative progression. This accumulation can be attenuated by ACAT1 inhibition, which promotes ABCA1-mediated cholesterol efflux via the LXR pathway [12,13]. Interestingly, these findings underscore the therapeutic relevance of lipid regulation in neurodegenerative contexts. Technological innovations such as protein misfolding cyclic amplification (PMCA) [14] have enabled cross-species screening of anti-prion compounds, identifying methylene blue as a potent inhibitor of prion replication [14,15] and validating PMCA’s utility in drug selectivity and structure–activity profiling. Moreover, in Parkinson’s disease models, high-throughput screening of marine and plant-derived fractions has uncovered bioactive molecules capable of alleviating mitochondrial stress, highlighting the untapped potential of natural products [16,17,18]. Among these, dehydrozingerone and its dimeric form have demonstrated notable neuroprotective effects in animal models, preserving dopaminergic neurons and motor function [19]. Similarly, ladostigil (*N*-propargyl-(3R)-aminoindan-5-yl)-*N*-propylcarbamate) [20,21] has shown promise in aging rats by modulating immune regulators such as TNFAIP3 and EGR1, thereby reducing memory decline and neuroinflammation [22]. Within the expanding realm of plant-derived therapeutics, *Syzygium aromaticum* (clove) [23,24] has emerged as a compelling candidate for intervention in Alzheimer’s disease, with its phytochemicals and amino acids contributing to antioxidant defense, anti-inflammatory activity, and neurotransmitter modulation [25,26]. Across various neurodegenerative conditions, research into adrenergic receptors suggests that repurposing existing drugs may offer new therapeutic avenues for Alzheimer’s disease [27], despite their incomplete mechanistic clarity. Other drug repurposing studies have highlighted the potential of atomoxetine [28] as part of neuroprotective strategies, including in the context of Alzheimer’s disease. Notably, a phase II clinical trial demonstrated excellent safety, tolerability, and target engagement in individuals with mild cognitive impairment, all advantages of repurposing a well-characterized FDA-approved medication [29]. Remarkably, in multiple sclerosis, immunoglobulin G and complement activation have been implicated in demyelination and neuronal damage, reinforcing the role of immune dysregulation in disease progression [30,31]. In parallel, spinocerebellar ataxia type 1 has inspired diverse therapeutic approaches, including genetic, pharmacological, and cellular investigations, aimed at preserving Purkinje cells and restoring motor coordination [32,33]. Meanwhile, nuclear medicine continues to refine TSPO radiotracers [34] for dementia imaging [35,36,37], with newer compounds addressing limitations in sensitivity and specificity (Table 1).

Altogether, these multidisciplinary efforts reflect a rapidly evolving landscape in neurodegenerative disease research. By integrating molecular insights, pharmacological innovation, and diagnostic precision, the field is advancing toward targeted interventions that address both etiological mechanisms and clinical manifestations. The convergence of natural product discovery, drug repurposing, and imaging technologies offers renewed promise for reducing disease burden and improving patient outcomes.

## Figures and Tables

**Table 1:** Overview of the pathological features, therapeutic strategies, and technological innovations herein discussed for some of the main neurodegenerative diseases.

Disease/Condition	Key Pathological Features	Therapeutic Strategies/Compounds
Alzheimer’s disease	Neuronal vulnerability, metabolic stress, proteotoxicity, protein misfolding	*Syzygium aromaticum* (clove), adrenergic receptor-targeting drugs, antioxidant and neurotransmitter support
Parkinson’s disease	Mitochondrial dysfunction, dopaminergic neuron loss	Dehydrozingerone and its dimer (in *Drosophila*), marine/plant-derived bioactives
Prion disorders	Protein misfolding, cross-species infectivity	Methylene blue
Multiple sclerosis	Myelin debris, cholesterol ester accumulation, immune dysregulation	ACAT1 inhibition, ABCA1-mediated cholesterol efflux via LXR pathway
Spinocerebellar ataxia type 1	Purkinje cell degeneration, motor coordination loss	Genetic, pharmacological, and cellular therapies
Aging brain	Lipid metabolism dysregulation, neuroinflammation, mitochondrial stress, protein misfolding	Natural compounds, synthetic agents, drug repurposing
Alzheimer’s disease	Cholesterol accumulation, immune activation	Ladostigil (modulates TNFAIP3 and EGR1), ACAT1 inhibition
	Neuronal vulnerability, metabolic stress, proteotoxicity, protein misfolding	*Syzygium aromaticum*, adrenergic receptor-targeting drugs, antioxidant and neurotransmitter support

For further details, readers are invited to consult the Special Issue “Biomolecular Approaches and Drugs for Neurodegeneration”, available at https://www.mdpi.com/journal/biomolecules/special_issues/O40SG4E9N5 (accessed on 30 August 2025).

## Abbreviations

The following abbreviations are used in this manuscript:

AD	Alzheimer’s disease
PD	Parkinson’s disease
MS	Multiple sclerosis
SCA1	Spinocerebellar ataxia type 1
ACAT1	Acyl-CoA:cholesterol acyltransferase 1
ABCA1	ATP-binding cassette transporter A1
LXR	Liver X receptor
PMCA	Protein misfolding cyclic amplification
TNFAIP3	Tumor necrosis factor alpha-induced protein 3
EGR1	Early growth response protein 1
TSPO	Translocator protein
IgG	Immunoglobulin G

## References

[1] Pathak N., Vimal S.K., Tandon I., Agrawal L., Hongyi C., Bhattacharyya S. (2022). Neurodegenerative disorders of alzheimer, parkinsonism, amyotrophic lateral sclerosis and multiple sclerosis: An early diagnostic approach for precision treatment. Metab. Brain Dis..

[2] Scheckel C., Aguzzi A. (2018). Prions, prionoids and protein misfolding disorders. Nat. Rev. Genet..

[3] Matilla-Dueñas A., Goold R., Giunti P. (2008). Clinical, genetic, molecular, and pathophysiological insights into spinocerebellar ataxia type 1. Cerebellum.

[4] Vicidomini C., Borbone N., Roviello V., Roviello G.N., Oliviero G. (2023). Summary of the Current Status of DNA Vaccination for Alzheimer Disease. Vaccines.

[5] Miller J.H., Das V. (2020). Potential for treatment of neurodegenerative diseases with natural products or synthetic compounds that stabilize microtubules. Curr. Pharm. Des..

[6] Rasool M., Malik A., Qureshi M.S., Manan A., Pushparaj P.N., Asif M., Qazi M.H., Qazi A.M., Kamal M.A., Gan S.H. (2014). Recent updates in the treatment of neurodegenerative disorders using natural compounds. Evid. Based Complement. Altern. Med..

[7] Leuci R., Brunetti L., Poliseno V., Laghezza A., Loiodice F., Tortorella P., Piemontese L. (2020). Natural compounds for the prevention and treatment of cardiovascular and neurodegenerative diseases. Foods.

[8] Falanga A.P., Piccialli I., Greco F., D’Errico S., Nolli M.G., Borbone N., Oliviero G., Roviello G.N. (2025). Nanostructural Modulation of G-Quadruplex DNA in Neurodegeneration: Orotate Interaction Revealed Through Experimental and Computational Approaches. J. Neurochem..

[9] Vicidomini C., Cioffi F., Broersen K., Roviello V., Riccardi C., Montesarchio D., Capasso D., Gaetano S.D., Musumeci D., Roviello G.N. (2019). Benzodifurans for biomedical applications: BZ4, a selective anti-proliferative and anti-amyloid lead compound. Future Med. Chem..

[10] Rahman M.M., Islam M.R., Supti F.A., Dhar P.S., Shohag S., Ferdous J., Shuvo S.K., Akter A., Hossain M.S., Sharma R. (2023). Exploring the therapeutic effect of neurotrophins and neuropeptides in neurodegenerative diseases: At a glance. Mol. Neurobiol..

[11] Chen X.-Y., Du Y.-F., Chen L. (2019). Neuropeptides exert neuroprotective effects in Alzheimer’s disease. Front. Mol. Neurosci..

[12] Huynh Krumeich T.N.P. (2024). Targeting the Cholesterol Storage Enzyme ACAT1/SOAT1 in Brain Cells and Mouse Model: A Novel Approach to Address Aging and APOE4-Related Neuroinflammation. Ph.D. Thesis.

[13] Zhou Y., Miles J.R., Tavori H., Lin M., Khoshbouei H., Borchelt D.R., Bazick H., Landreth G.E., Lee S., Fazio S. (2019). PMP22 regulates cholesterol trafficking and ABCA1-mediated cholesterol efflux. J. Neurosci..

[14] Saá P., Cervenakova L. (2015). Protein misfolding cyclic amplification (PMCA): Current status and future directions. Virus Res..

[15] Cavaliere P., Torrent J., Prigent S., Granata V., Pauwels K., Pastore A., Rezaei H., Zagari A. (2013). Binding of methylene blue to a surface cleft inhibits the oligomerization and fibrillization of prion protein. Biochim. Biophys. Acta (BBA) Mol. Basis Dis..

[16] Silva J., Alves C., Soledade F., Martins A., Pinteus S., Gaspar H., Alfonso A., Pedrosa R. (2023). Marine-derived components: Can they be a potential therapeutic approach to Parkinson’s disease?. Mar. Drugs.

[17] Huang C., Zhang Z., Cui W. (2019). Marine-derived natural compounds for the treatment of Parkinson’s disease. Mar. Drugs.

[18] Fakhri S., Abdian S., Zarneshan S.N., Akkol E.K., Farzaei M.H., Sobarzo-Sánchez E. (2021). Targeting mitochondria by plant secondary metabolites: A promising strategy in combating Parkinson’s disease. Int. J. Mol. Sci..

[19] Kesharwani A., Sree B.K., Singh N., Gajbhiye R.L., Murti K., Peraman R., Pandey K., Limoli C.L., Velayutham R., Parihar V.K. (2025). Dehydrozingerone Improves Mood and Memory in Diabetic Mice via Modulating Core Neuroimmune Genes and Their Associated Proteins. ACS Pharmacol. Transl. Sci..

[20] Weinstock M. (2025). Role of Oxidative Stress and Neuroinflammation in the Etiology of Alzheimer’s Disease: Therapeutic Options. Antioxidants.

[21] Weinreb O., Amit T., Bar-Am O., BH Youdim M. (2012). Ladostigil: A novel multimodal neuroprotective drug with cholinesterase and brain-selective monoamine oxidase inhibitory activities for Alzheimer’s disease treatment. Curr. Drug Targets.

[22] Zohar K., Lezmi E., Reichert F., Eliyahu T., Rotshenker S., Weinstock M., Linial M. (2025). Temporal shifts in microRNAs signify the inflammatory state of primary murine microglial cells. Int. J. Mol. Sci..

[23] Cortés-Rojas D.F., de Souza C.R.F., Oliveira W.P. (2014). Clove (Syzygium aromaticum): A precious spice. Asian Pac. J. Trop. Biomed..

[24] Valarezo E., Ledesma-Monteros G., Jaramillo-Fierro X., Radice M., Meneses M.A. (2025). Antioxidant Application of Clove (Syzygium aromaticum) Essential Oil in Meat and Meat Products: A Systematic Review. Plants.

[25] Sargsyan T., Simonyan H.M., Stepanyan L., Tsaturyan A., Vicidomini C., Pastore R., Guerra G., Roviello G.N. (2025). Neuroprotective Properties of Clove (Syzygium aromaticum): State of the Art and Future Pharmaceutical Applications for Alzheimer’s Disease. Biomolecules.

[26] Uchewa O.O., Egwuagu C.B., Ibegbu A.O. (2025). Clove oil as a neuromodulator in environmental cadmium cognitive impairment on the prefrontal cortex of Wistar rats. J. Trace Elem. Miner..

[27] Wang A., Since M., Dallemagne P., Rochais C. (2025). Implication of Central β2 Adrenergic Receptor for the Development of Novel Drugs Against Alzheimer’s Disease. Arch. Der Pharm..

[28] Roviello G., Cioffi C., Moccia M., Gillick-Healy M.W., Kelly B.G., Adamo M.F.A. (2025). Synthesis of active pharmaceutical ingredient atomoxetine via desulfurative halogenation. Tetrahedron Lett..

[29] Levey A.I., Qiu D., Zhao L., Hu W.T., Duong D.M., Higginbotham L., Dammer E.B., Seyfried N.T., Wingo T.S., Hales C.M. (2022). A phase II study repurposing atomoxetine for neuroprotection in mild cognitive impairment. Brain.

[30] Saez-Calveras N., Stuve O. (2022). The role of the complement system in Multiple Sclerosis: A review. Front. Immunol..

[31] Garton T., Gadani S.P., Gill A.J., Calabresi P.A. (2024). Neurodegeneration and demyelination in multiple sclerosis. Neuron.

[32] Ripolone M., Lucchini V., Ronchi D., Fagiolari G., Bordoni A., Fortunato F., Mondello S., Bonato S., Meregalli M., Torrente Y. (2018). Purkinje cell COX deficiency and mtDNA depletion in an animal model of spinocerebellar ataxia type 1. J. Neurosci. Res..

[33] Tejwani L., Lim J. (2020). Pathogenic mechanisms underlying spinocerebellar ataxia type 1. Cell. Mol. Life Sci..

[34] Pulagam K.R., Colás L., Padro D., Plaza-García S., Gómez-Vallejo V., Higuchi M., Llop J., Martín A. (2017). Evaluation of the novel TSPO radiotracer [18F] VUIIS1008 in a preclinical model of cerebral ischemia in rats. EJNMMI Res..

[35] Salerno S., Viviano M., Baglini E., Poggetti V., Giorgini D., Castagnoli J., Barresi E., Castellano S., Da Settimo F., Taliani S. (2024). TSPO radioligands for neuroinflammation: An overview. Molecules.

[36] Uzuegbunam B.C., Rummel C., Librizzi D., Culmsee C., Hooshyar Yousefi B. (2023). Radiotracers for Imaging of Inflammatory Biomarkers TSPO and COX-2 in the Brain and in the Periphery. Int. J. Mol. Sci..

[37] Herholz K., Carter S., Jones M. (2007). Positron emission tomography imaging in dementia. Br. J. Radiol..

